# Equal Graph Partitioning on Estimated Infection Network as an Effective Epidemic Mitigation Measure

**DOI:** 10.1371/journal.pone.0022124

**Published:** 2011-07-22

**Authors:** Jeremy Hadidjojo, Siew Ann Cheong

**Affiliations:** Division of Physics and Applied Physics, School of Physical and Mathematical Sciences, Nanyang Technological University, Singapore, Republic of Singapore; National Institute of Environmental Health Sciences, United States of America

## Abstract

Controlling severe outbreaks remains the most important problem in infectious disease area. With time, this problem will only become more severe as population density in urban centers grows. Social interactions play a very important role in determining how infectious diseases spread, and organization of people along social lines gives rise to non-spatial networks in which the infections spread. Infection networks are different for diseases with different transmission modes, but are likely to be identical or highly similar for diseases that spread the same way. Hence, infection networks estimated from common infections can be useful to contain epidemics of a more severe disease with the same transmission mode. Here we present a proof-of-concept study demonstrating the effectiveness of epidemic mitigation based on such estimated infection networks. We first generate artificial social networks of different sizes and average degrees, but with roughly the same clustering characteristic. We then start SIR epidemics on these networks, censor the simulated incidences, and use them to reconstruct the infection network. We then efficiently fragment the estimated network by removing the smallest number of nodes identified by a graph partitioning algorithm. Finally, we demonstrate the effectiveness of this targeted strategy, by comparing it against traditional untargeted strategies, in slowing down and reducing the size of advancing epidemics.

## Introduction

Understanding and containing the spread of an infectious disease has always attracted a lot of interest from the scientific community, and even more so after the recent SARS and H1N1 outbreaks. Besides their social and healthcare impacts, severe infectious disease outbreaks also present an important burden to the economy through the decrease in productivity and high cost of treatment. As denser populations promote faster spreading, this problem can only grow in severity and magnitude with the increasing world population density. Motivated by this, many works have been done to predict [1], [2] or even contain [3]–[6] the spread of severe epidemics. In spite of these efforts, effective control of infectious disease outbreaks continues to elude us.

A recent important advancement in the field is the application of network theory to study epidemic dynamics. Network-based models have been shown able to accurately explain complex phenomena in terms of the relatively-simple interactions between its small constituents, and are therefore broadly applicable to various different fields [7]. In the area of infectious diseases, numerous studies have been done on modeling epidemic using a network approach - mainly covering sexually-transmitted infections [8]–[11], respiratory and flu-like diseases [2], and general features of infectious disease dynamics [12]–[17].

From the network point of view, we speculate that different infectious diseases might have very similar infection networks if they share the same mode of transmission. In particular, we believe that severe respiratory infections such as H1N1 and SARS share an infection network similar to that of the less severe common cold. Hence, infection network inferred from the latter can be useful in controlling rare outbreaks of the former. Estimating the infection network from common infections has the advantage of a large volume of daily incidences. As we will show in the Results section, the volume of incidences data gathered is critical in getting accurate estimation of the network.

Traditional epidemic intervention procedures, such as quarantine and other social distancing measures, involve weakening or cutting links around the infected nodes. However, these procedures are not systematic from the network point of view. A more effective intervention strategy would employ understanding the ‘shape’ of the infection network and applying that knowledge to efficiently tear the network apart. This can be done by targeting nodes that play important roles in the network (i.e. the ‘hubs’), and the ‘backbones’ connecting one hub to another.

Based on the above ideas, we proposed a targeted method to effectively contain infectious disease epidemics. This strategy involves estimating the infection network of a severe disease using incidences data from common infections sharing the same infection network, and fragmenting the network into disconnected pieces using a graph partitioning method. To test the proposed strategy in principle, we first generate artificial social networks of different sizes but with roughly the same clustering characteristics to serve as our infection networks. Then we simulate multiple SIR epidemics on the networks to play the role of common infections circulating in society. We apply censorships on the incidence data collected to emulate the low reporting rates of common infections, and use the censored incidences to construct estimates of the original infection networks. To mitigate new epidemics, we fragment the estimated infection networks. To do this, we apply a graph partitioning method on the estimated networks to identify the smallest sets of nodes that, when removed, will efficiently break the networks up into isolated pieces. Finally, we evaluate the effectiveness of this targeted strategy by comparing against traditional untargeted methods. While each of the problems has been studied independently (see for example [8], [18] for network reconstruction and [19]–[22] for graph partitioning), to the best of our knowledge no work has been done applying both methods to control epidemics.

## Methods

At this point in time, we know of no available databases of common infections that (1) are comprehensive enough for reconstructing the infection network and (2) have relevant on-going epidemics to test the proposed strategy. Hence, we used computer simulations to study the proposed method.

### 2.1 Artificial Infection Network Generation

Many naturally-occurring networks like the Internet, the World Wide Web, and biological networks are scale-free and are thus well described by Barabasi’s preferential attachment model [23]. Others argued, however, that social networks are different as they show strong clustering of nodes (also called community structures) and their degree distributions are not power laws [24]. To specifically reproduce the community structures seen in social and social-like networks, Holme and Kim modified the preferential attachment model to incorporate clustering [25]. Newman *et al.*, on the other hand, started out with random graphs and progressively build the social-like degree distribution [26], whereas Boguna *et al.* and Jin *et al.* proposed friendship-formation dynamics models to generate social-like networks from scratch [27], [28].

For our proof-of-concept study, we generate social-like networks to act as our infection networks. We follow the three intuitive rules described by Jin, Girvan, and Newman (JGN) in Ref. [28]: (1) the probability of two individuals meeting is high if they have one or more mutual friends, and low otherwise; (2) the friendship between two individuals is reinforced by regular meetings, but decays with time if they rarely meet; and (3) there is a maximum number of friends one can have.

Following the discrete algorithm presented in Ref. [28], we first start with a random network of *N* nodes and *m* links. The average degree of the network (average number of links per node) is given by 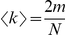. At each time step, we select *n_m_* pairs of nodes that have at least one mutual friend. This is done by first picking *n_m_* intermediate nodes at random, before choosing two neighbours of each to become the pairs. In addition to this, we choose *n_r_* other pairs of nodes uniformly at random, with *n_m_*>*n_r_*. For every pair that is not already connected, we form a new link between them provided that both nodes have not reached the maximum number of friends *L*. At the end of each time step, we randomly break *r* existing links to simulate the friendship decay. We then calculate the clustering coefficient *c* of the network using the method by Schank and Wagner [29]. After the clustering coefficient stops increasing and only fluctuates about a time-independent long-run average, we say the network has converged and stop the simulation. As expected, the clustering coefficients of the converged networks are much higher than 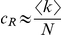 for random networks with the same *N* and *<k>*.

In generating the infection networks, we set *L* = 50 as the maximum number of friends a node can have. To produce high clustering coefficients, we ensure that the mutual friend formation is dominant over the random friend formation by choosing *n_m_* = 400 and *n_r_* = 100. For simplicity, we choose *r* = *n_r_*+*n_m_* = 500 such that the total number of links (hence the average degree *<k>*) of the initial random network remains more or less constant. This way, we can generate social-like infection networks with arbitrary size and average degree by simply adjusting *N* and *<k>* of the initial random network to the desired values.

### 2.2 SIR Epidemics

After generating the infection network, we simulate *S* susceptible-infected-recovered (SIR) epidemics. To start the epidemic, we initialize the network so that all nodes are susceptible. One random node is then infected to act as seed of the epidemic. At each time step, every infected node will transmit the disease to its susceptible neighbours with probability *q*. After a certain time interval *t_R_,* the infected nodes will recover and become immune to subsequent infections. This way, the number of infected nodes grows from the single seed, peaks, and thereafter decreases as more and more nodes recover and become immune. When there are no more infected nodes in the network, the epidemic ends and all nodes are reset to the susceptible state for simulating the next epidemic.

In this SIR model, the probability *q* of infecting susceptible neighbours reflects the characteristic infection rate of the particular disease over the simulation time step *Δt*. For a given time step size *Δt*, a more infectious disease will have a larger *q*, whereas a less infectious disease will have a smaller *q*. For a given infectious disease, *q* will be smaller for a smaller *Δt*, and larger for a larger *Δt*. The simulation time step *Δt* itself is chosen, for simplicity, to be roughly equal to the typical incubation and recovery period of the disease (about 3–5 days for common cold). This implies that *t_R_≈Δt,* i.e. the infected nodes recover after one simulation time step.

When running the simulations, we find that the infection rate *q* must be greater than a certain minimum value *q_min_* for the epidemics to be self-sustaining and cover a large fraction of the network. We also observe that *q_min_* depends on the network’s average degree *<k>*. A less-connected network with smaller average degree *<k>* requires a larger value of *q_min_* to sustain an epidemic, as compared to a more highly-connected network with higher *<k>*. Hence, we fine tune the infection probability *q* so that 50%–70% of the nodes are infected in one epidemic simulation. We find that *q_min_* ≈ 0.80 in the case of *<k>* = 3, *q_min_ ≈* 0.20 for *<k>* = 10, and *q_min_ ≈* 0.08 for *<k>* = 20. Finally, we note that while it is relatively easy to infect the majority of the nodes in the network with high *<k>*, infecting all nodes is extremely hard. We need *q* to be very close to one before the epidemic infects the entire network.

### 2.3 Censorship and Network Estimation

When collecting incidence data of common and mild diseases, we expect low incidence reporting rates because: (1) some infected individuals do not show symptoms (sub-clinical cases); and (2) some others choose not to consult doctors despite showing symptoms (unreported cases). In countries where consultation fees are high, we expect the latter to be dominant. Fortunately, the low reporting rates are compensated by the large volume of daily incidences generated by such diseases. We also note that voluntary reporting rates in Singapore are relatively high because of the low consultation fees, making the strategy for estimating the infection network presented below attractive.

To simulate different reporting rates, we censor the incidence data before using them to estimate the infection network. Here, the censor rate 0<*C*<1 is defined as the fraction of incidence data discarded randomly, and hence not used in the network estimation. For our study, we vary *C* from 50% to 85%.

**Figure 1:**
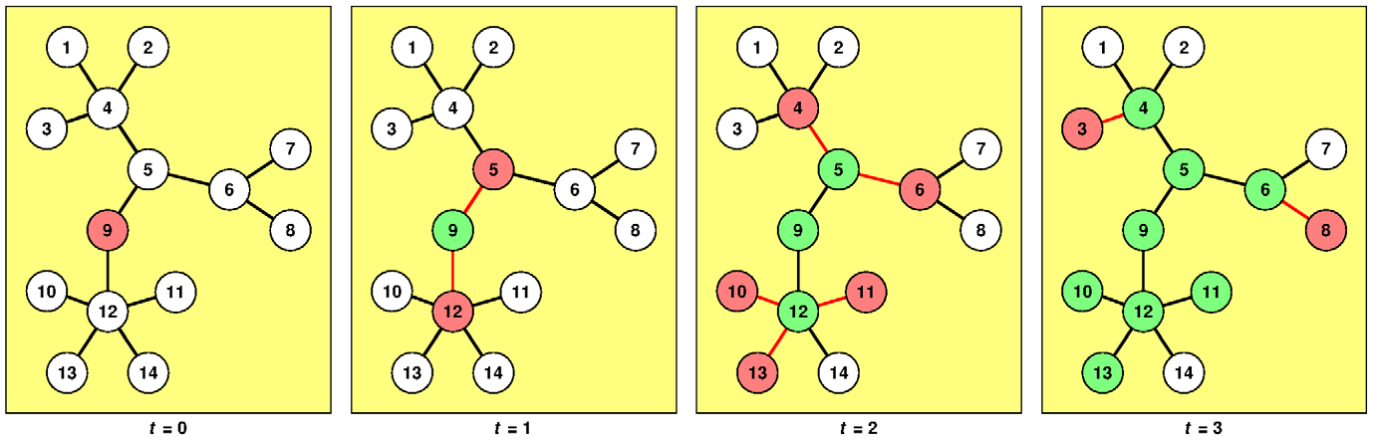
An SIR epidemic spreading through a small network of *N* = 14 nodes. The epidemic starts on node 9 at *t* = 0. Thereafter, nodes 5 and 12 are infected at *t* = 1 by node 9, which recovers and become immune to subsequent infection. At *t* = 2, nodes 4 and 6 are infected by node 5 (which recovers), while nodes 10, 11, and 13 are infected by node 12 (which recovers). Following this, at *t* = 3, node 3 is infected by node 4 (which recovers), while node 8 is infected by node 6 (which recovers). Nodes 10, 11, and 13 recover without infecting any other nodes. Finally, at *t* = 4 (not shown), nodes 3 and 8 recover and the epidemic ends. In this figure, susceptible nodes are white, infected nodes are red, recovered nodes are green, and links responsible for the transmission are colored red for the time step they are activated.

Our network estimation algorithm is essentially a modified JGN algorithm. To estimate the unknown infection network from the incidence data, we note that all newly-infected individuals must have been infected by individuals that were infected one time step back, as shown in Figure 1. For the epidemic shown in Figure 1, the incidences are {9}, {5, 12}, {4, 6, 10, 11, 13}, {3, 8} at time steps *t* = 0, 1, 2, 3 respectively. Suppose some of these incidences were censored, and we ended up with the censored incidences {9}, {5}, {4, 11, 13}, {8} at time steps *t* = 0, 1, 2, 3 respectively. Based on these censored incidences, we then draw a tentative link between nodes 9 and 5, because the infection of node 9 precedes the infection of node 5. We then draw tentative links between the pairs (5, 4), (5, 11), (5, 13) because the infection of node 5 precedes the infection of nodes 4, 11, 13. Finally, we draw a tentative link between the pairs (4, 8), (11, 8), (13, 8), because the infection of nodes 4, 11, 13 precedes the infection of node 8.

As we can see, only the links (4, 5) and (5, 9) are correctly estimated. The rest of the estimated links are all wrong, partly because of the algorithm, and partly because of the censorship. To improve the accuracy of our estimated network, all tentative links are introduced with a weight of *w* = 1.00. Thereafter, the weight of all links decay by *Δw* = −0.01 every time step. Tentative links are removed when their weights fall to zero. In the above example, the links (5, 9), (4, 5), (5, 11), (5, 13), (4, 8), (8, 11), (8, 13) are introduced at *t* = 1, 2, 2, 2, 3, 3, 3, and so when the epidemics end at *t* = 4, their weights would be 0.97, 0.98, 0.98, 0.98, 0.99, 0.99, 0.99 respectively. We then repeat the estimation over more SIR epidemics. In subsequent epidemics, if an existing estimated link is reactivated, it is reinforced by adding *Δw* = 1.00 to its weight. We expect the algorithm to produce mostly wrong links at first. However, as the weights are decaying with time, most of the wrongly-estimated links will disappear at the end, because they will not consistently appear in the estimation process. On the other hand, correctly-estimated links tend to get constantly reinforced over multiple epidemics. Hence, we expect the population of correctly-estimated links to increase over time. More importantly, we note that existing estimation can always be further refined by incorporating new incidence data as and when they become available.

### 2.4 Network Partitioning

From the network point of view, efficient epidemic mitigation can be achieved by identifying and targeting the most densely-connected nodes (also called ‘hubs’) in the infection network. However, targeting only hubs in the infection network may not be the most efficient mitigation strategy, because a strongly clustered network may not break up into fragments after these are removed. Since we want to fragment the infection network to stop the epidemic from spreading across the entire network, we perform equal graph partitioning (EGP) [22] on the estimated network. EGP identifies the smallest set of nodes which, when removed, will break the network into isolated chunks of roughly equal sizes.

To start the EGP, we first randomly assign every node into two groups A and B. We then move all nodes in A that is connected to B, and all nodes in B that is connected to A, into a third group C (also called the separator group). Once this is done, there are no nodes in A that is connected directly to B and vice versa. Following this we minimize the size of group C by swapping nodes in C with those in A and B, without introducing direct connections between A and B. A swap is accepted if it results in a smaller group C. We perform repeated swaps until no further improvements can be made. If we then remove this optimal set of nodes in C, the network will be efficiently broken into two disconnected chunks. When necessary, the resulting chunks can be further fragmented into even smaller pieces by applying EGP recursively.

## Results

### 3.1 Preliminary Study

For our preliminary study, we tested our estimation algorithm on a small social network with *N* = 1,000 nodes, average degree *<k>* = 3, and clustering coefficient *c* = 0.43 (random network with the same *N* and *<k>* would have clustering coefficient c* ≈ *<k>/N* = 0.003). Besides allowing shorter simulations on a desktop computer, this smaller network has the advantage that the entire network, together with the simulated epidemics, can be easily visualized (see Videos S1 and S2).

**Figure 2:**
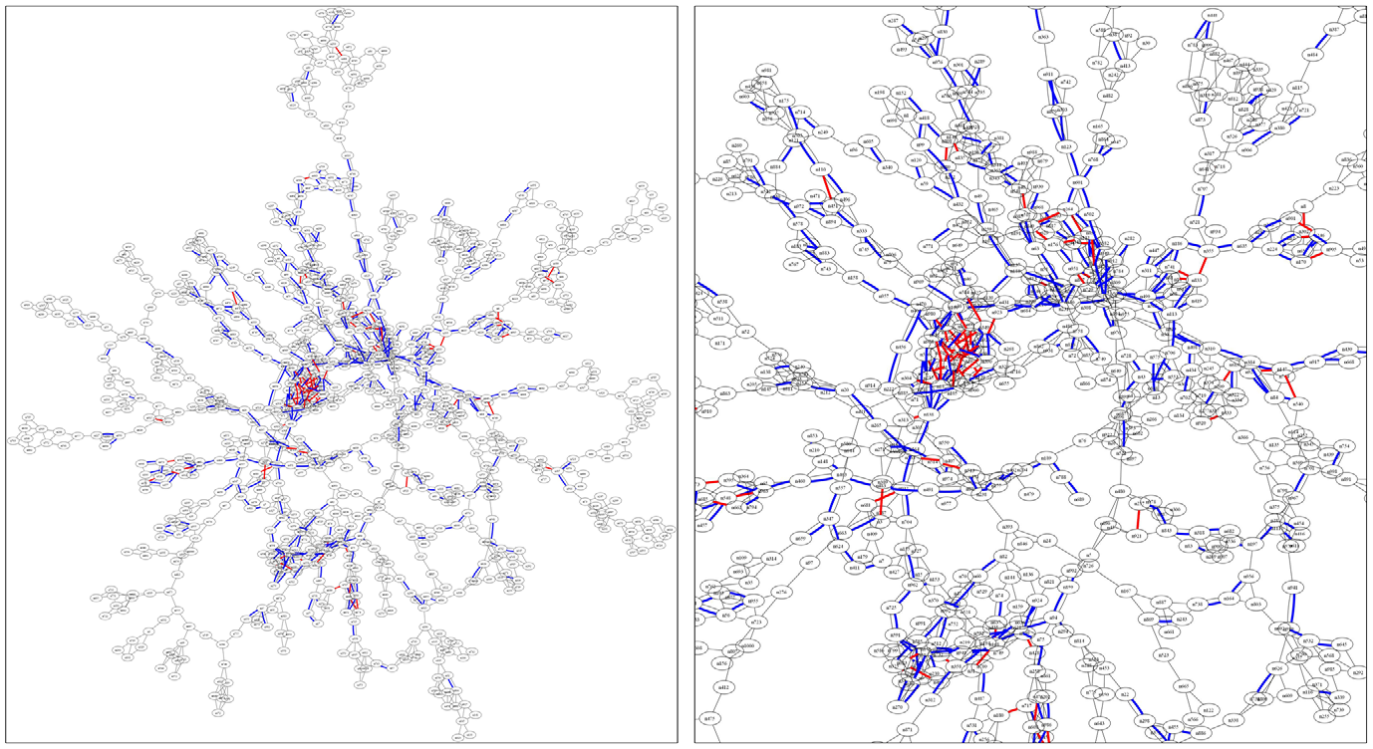
Estimation of a small infection network, shown superimposed onto the original network. Blue lines represent correctly estimated links, whereas red lines represent incorrectly estimated links. Full view of the network (left) and magnified view around the center (right) are shown, highlighting the highly-connected nodes (hubs) and backbones connecting them. The estimation is based on 100 SIR epidemics and 70% censor rate. The original infection network has 1,000 nodes, 1,584 links, and average degree <*k*> = 3. About 60% of total links are estimated with 79% accuracy.

We simulated *S* = 100 epidemics and censored 70% of the incidence data before estimating the infection network. The estimated network is shown in Figure 2, superimposed onto the original network. The reconstruction is highly accurate in this test case. From the total 1,584 links in the original network, the algorithm estimated 738 links correctly and made only 196 wrong estimates, resulting in 79% estimation accuracy. More importantly, the estimation found mostly links between highly connected ‘hubs’ that form the ‘backbones’ of the network. These backbones play an important role in disease transmissions as they link one highly-connected cluster (community) to another throughout the network.

After estimating the infection network, we performed EGP and found a set of 67 nodes that will efficiently fragment the estimated network. We then immunized the same 67 nodes on the actual infection network. To evaluate the effectiveness of the method, we picked 10 random seeds and ran 500 SIR epidemics each on the immunized network to find the average number of infected nodes as a function of time. Using the same seeds, we also ran 500 SIR epidemics each on the original infection network and found that EGP immunization reduced the average size of the epidemics by 58%–88% and lowered the average peak incidence rate by 51%–68% (see Videos S3, S4, S5, and S6).

### 3.2 Systematic Study

Motivated by the promising result of small network estimation, we performed systematic studies of larger networks to see how well these can be estimated. We varied the number of estimated links *n*, the censor rate *C*, the size of the network *N*, the number of SIR epidemics *S* used for estimation, and the average degree of the network *<k>*. The same clustering coefficient of *c* = 0.05 is maintained across the networks to ensure meaningful comparisons.

#### 3.2.1 Accuracy versus number of estimated links

**Figure 3:**
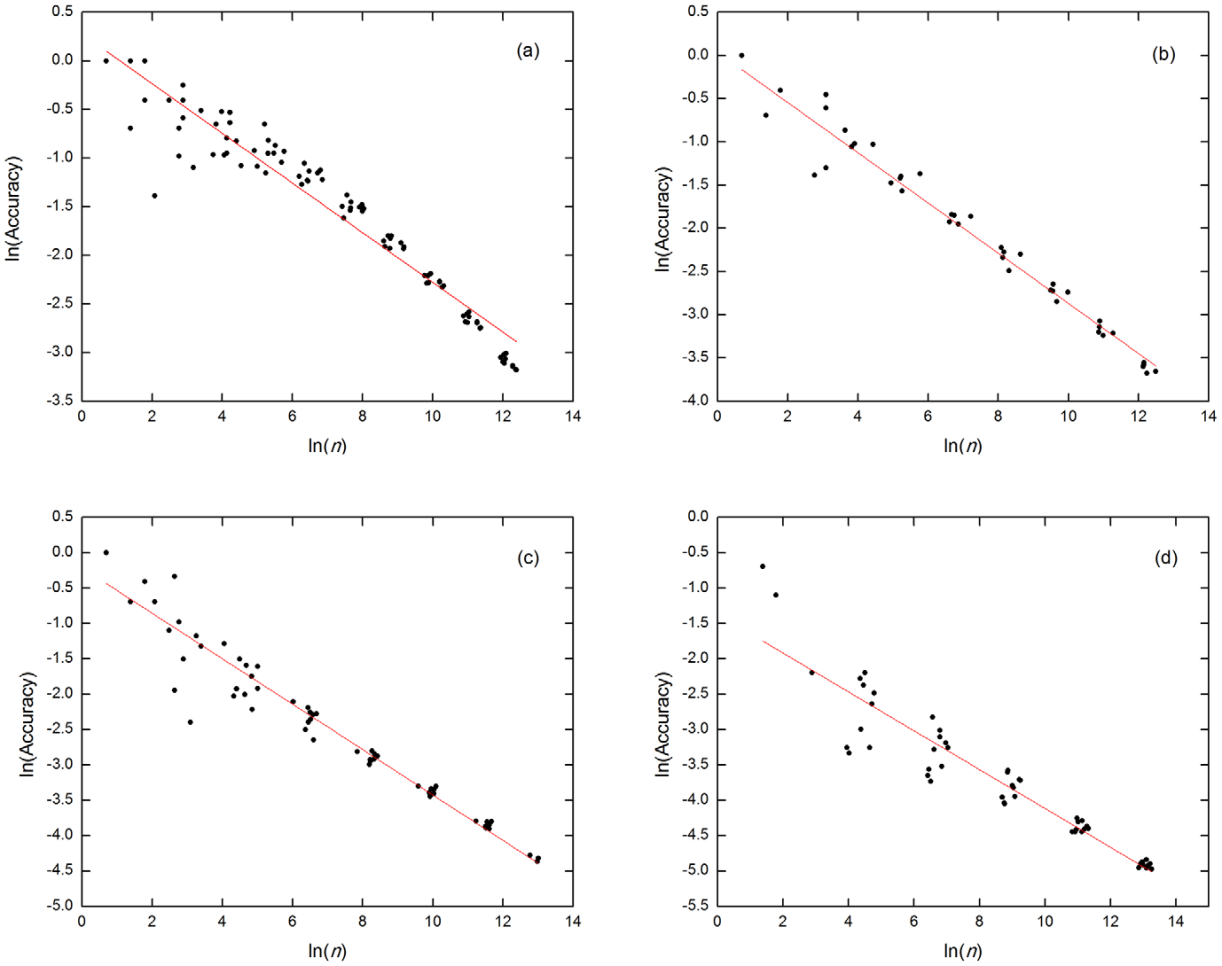
Log-log plots of estimation accuracy as a function of the number of estimated links *n*. The censor rates used are (a) 50%, (b) 60%, (c) 70%, and (d) 80%. Red lines show the best linear fits of the data (used to extrapolate and compare accuracies across different censor rates in Figure 3). The slope *a* and y-intercept *b* of the linear fits are: (a) *a* = (−0.256 ± 0.006) and *b* = (0.279 ± 0.046); (b) *a* = (−0.291 ± 0.008) and *b* = (0.038 ± 0.060; (c) *a* = (−0.322 ± 0.008) and *b* = (−0.211 ± 0.064); (d) *a* = (−0.275 ± 0.014) and *b* = (−1.368 ± 0.126). The decay exponents (slope in log-log plot above) are found to be between −0.24 and −0.32, thus implying that the accuracy actually falls off very slowly with the number of estimated links *n*. All estimations are based on 100 SIR epidemics. The actual infection network has *N* = 10,000, *<k>* = 10, and *c* = 0.05.

In a given estimation, there is a trade-off between accuracy and number of estimated links *n*. At the end of the estimation, frequently reinforced links (likely to be the correct ones) will have weights much higher than those infrequently reinforced links (likely to be the wrong ones). To determine which tentative links we would accept as estimated links, we set a cut-off value *w_C_* for the weight. Links with weights above *w_C_* will be accepted as estimated links, otherwise they are rejected. If *w_C_* is large, only a few estimated links are accepted, and most of these will be correct estimates. On the other hand when *w_C_* is small, more estimated links are accepted but a larger fraction of these will be wrong. Our simulation results confirmed this and showed that the accuracy falls off very slowly as a power law in the number of estimated links *n*, with power-law exponents much smaller than 1 (Figure 3).

#### 3.2.2 Accuracy versus censor rate *C*

**Figure 4:**
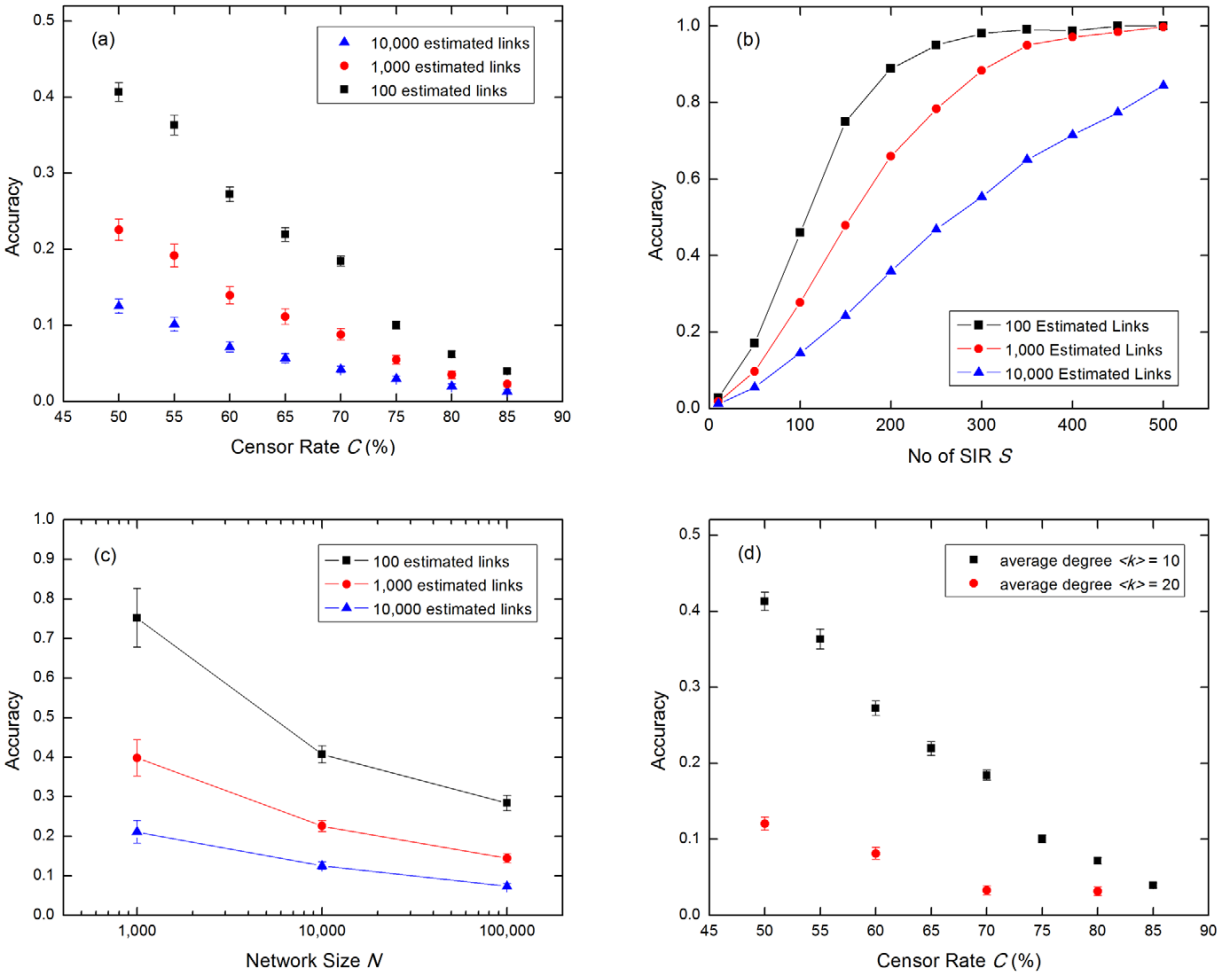
Systematic studies of the estimation accuracy. We plot the estimation accuracy versus: (a) censor rate *C*, (b) number of SIR epidemics *S*, (c) network size *N*, and (d) censor rate for different value of average degrees *<k>.* Since we cannot precisely stop at arbitrary number of estimated links by adjusting *w_c_* alone, we extrapolate from the linear fits (Figure 2) to obtain and compare accuracies of *n* = 100, 1,000, and 10,000 estimated links in figures above. The error bars are calculated from the error of fitting parameters, where appropriate. The graphs are obtained with the following simulation parameter: (a) *N* = 10,000, *<k>* = 10, and *S* = 100; (b) *N* = 10,000, *<k>* = 10, and *C* = 50%; (c) *<k>* = 10, *S* = 100, and *C* = 50%; (d) *N* = 10,000 and *S* = 100. All networks have *c* = 0.05.

As common infections are typically under-reported, it is important to study how the estimation accuracy varies with censor rate. For a fixed network with *N* = 10,000 nodes and *<k>* = 10, we simulated *S* = 100 SIRs and applied various censor rates *C* from 50% to 85%. The estimation accuracy is found to decrease as *C* increases (Figure 4a). This is expected, as lower censor rate results in more data available for estimation and thus improves the accuracy.

#### 3.2.3 Accuracy versus number of SIR *S*

Incidence data from a single epidemic will not provide a good picture of the network, because of censorship and history dependence of the epidemic. For different seeds, the epidemic progresses along different transmission routes. Thus, even if we could track all incidences for a given epidemic we are seeing an incomplete snapshot of the infection network. With censorship, this snapshot is even more incomplete. This is why we need to accumulate information from successive epidemics to make consistent and reliable estimates of the network links that participate in the transmission. In Figure 4b, we show the estimation accuracy as a function of the number of different SIR epidemics *S* used in the estimation with 50% censor rate. We found the accuracy to improve with *S* initially before saturating to 100% as more incidence data becomes available. It thus appears that faithful estimation of the infection network is asymptotically possible.

#### 3.2.4 Accuracy versus number of nodes *N* and average degree *<k>*

Naturally, we expect larger and highly-connected networks to be more difficult to estimate. To study how the estimation accuracy varies with *N*, we performed estimation on networks with three different sizes *N* = 1,000, 10,000, and 100,000 using data from *S* = 100 epidemics. We also compared the estimation accuracies for networks with *N* = 10,000 nodes and two different average degrees *<k>* = 10 and 20. In Figure 4c–d, we show that the accuracy falls with increasing network size *N* and increasing average degree *<k>,* as expected.

Summarizing the results of the systematic studies, the estimation accuracy for a given infection network depends strongly on the censor rate, the number of epidemics over which incidence data is accumulated, and the average degree of the network. The accuracy falls with increasing censor rate, network size, and average degree, but improves over time as more incidence data from new epidemics become available.

### 3.3 Graph Partitioning

#### 3.3.1 Pre-epidemic EGP

Since the preliminary EGP result in Section 3.1 is encouraging, we tried EGP mitigation on the larger networks despite the poor estimation accuracy.

**Figure 5:**
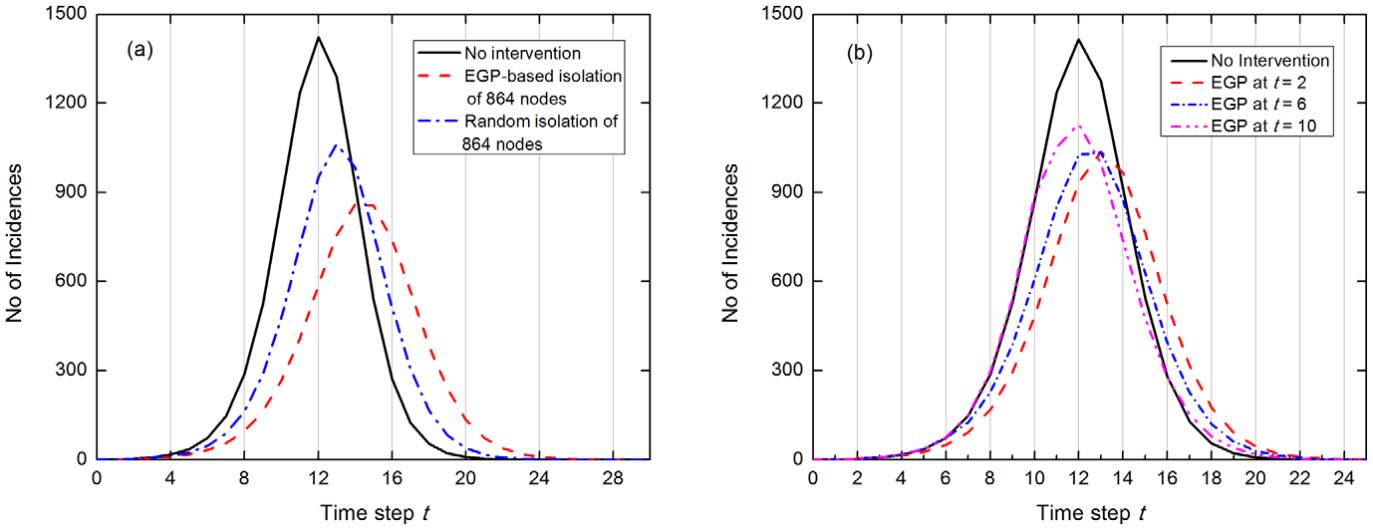
Effectiveness of EGP-based intervention. (a) 864 nodes identified through EGP on the estimated network were isolated from the actual network before the start of epidemics. The average infection rate after EGP-based mitigation is compared against average infection rates without mitigation and with random isolation of 864 nodes. The average number of infected nodes per epidemic after EGP-based mitigation is decreased to 6,345 nodes, as compared to 7,881 and 6,747 average infected nodes for no mitigation and after random isolation, respectively. (b) The same EGP-based intervention as in (a), except done at different times after the epidemics have started. The EGP-based strategy is surprisingly effective on controlling epidemics, with EGP applied even at 10 time step after the start of the epidemics still effective in reducing peak infection rates and total number of infected nodes.

We started by estimating an infection network (*N* = 10,000, *<k>* = 10, and a total of 50,126 links) using incidence data from 100 SIR epidemics and 50% censor rate. The estimated network has a total of 2,561 nodes and 6,467 estimated links. 1,074 of these estimated links are correct (16.6% accuracy). Next, we applied EGP to this estimated network (*not* to the actual infection network) and obtained a set of 864 nodes that will efficiently fragment the estimated network. We then immunized these 864 nodes on the actual infection network to test how well this will mitigate subsequent epidemics. To obtain a non-targeted intervention benchmark for comparison, we also randomly removed 864 nodes in the actual infection network. We then ran 100 SIR epidemics each on the two immunized networks and plot the average incidence rate as a function of time, as shown in Figure 5a.

Considering the estimated network represents only a small fraction (0.13) of the actual number of links, and only 16.6% of the estimated links are correct, the EGP-based mitigation is surprisingly effective in both slowing down the progress and reducing the peak incidence rate of the outbreaks. We show that systematic isolation of only 8.6% of the total population reduced the peak infection rate by about 40% and delayed the peak infection time by about 20%, as compared to the unmitigated case. More importantly, even though random isolation also reduced and delayed the peak, the targeted intervention is still significantly better.

The 864 separator nodes identified by EGP are clearly special on the estimated network (and thus also on the actual network). However, all the 2,561 nodes identified in the estimation process are important, because they are on the transmission paths for nearly all epidemics. As suggested by the referee, removing all these nodes may bring the infection network below its percolation threshold, thereby completely stopping all epidemics. To test this idea, we isolated the 2,561 nodes on the actual network and ran 100 SIR simulations. We found that in about 40 simulations, the ‘epidemics’ infected only 2–5 nodes and died off very quickly after 1 or 2 time steps after starting. For the other 60 simulations, we observe very slowly circulating epidemics that lasted more than 60 time steps and infected 2,580 nodes on average, with the number of incidences reaching a peak of 116 cases 28 time steps after the start.

#### 3.3.2 In-epidemic EGP

The results above were obtained assuming we can partition the network before the start of each epidemic. Since it is more realistic to assume we intervene only after an outbreak has been detected, we also investigated the effectiveness of the EGP intervention enforced at different times during the epidemic (Figure 5b). Our simulation shows that the strategy is still very effective even when it is applied as late as 10 time steps (roughly 30 days) after the start, just 2 time steps away before the peak. The peak infection rate is reduced by about 20% and total number of cases by 12%, as compared to the unmitigated case. Unfortunately, the time of maximum infection rate is not delayed when EGP is applied at *t* = 10, and is only delayed by about one time step when intervention is applied at *t* = 2 and *t* = 6 time steps after the start.

## Discussion

### 4.1 Infection network estimation

Based on the results reported in Section 3.3, we have shown that the proposed intervention based on estimated infection networks is very effective in limiting the scale and slowing down the spread of infectious disease epidemics. However, as discussed in Section 3.2, the estimated infection networks are not always accurate, especially when the proportion of unreported incidences is high. Figure 4a suggests that the estimation accuracy goes to zero at censor rate *C_T_*<100%. This implies that it might be difficult to do network estimation in the US, where censor rates for mild illnesses are close to 100% because of the expensive medical insurance and co-payments. However, we stand a better chance of making the scheme work in Singapore as the low medical cost and the necessity to obtain medical certificates to stay away from work promote doctor consultations even for mild symptoms. To further improve the accuracy, the incidence data can be easily complemented with other types of relevant data. For instance, cell-phone and GPS co-location information can be used to rule out a large proportion of tentative links connecting pairs of individuals that have never been found in the same location over the infection time step of about three days. This will reduce the rate of forming false links significantly, thus improving the accuracy of the estimation.

Real infection networks are also likely to be dynamic and changing with time. There are seasonal variations to the infections, and also seasonal variations to social mobility patterns. For example, most temperate countries have an annual flu season that peaks during winter [30]–[32], whereas tropical countries experience multiple flu seasons each year [33]. School holidays in June and December also change the social dynamics drastically, as students start hanging out more in shopping centers and families go on vacations [34], [35]. With real data, what we are estimating is effectively the infection network averaged over multiple epidemiological and social seasons. With enough data, it might also be possible to estimate these seasonal infection networks, and clearly interventions and mitigations might become more effective with the added knowledge of how the network varies with time. However, we believe that even with the averaged infection network, interventions and mitigations will still be effective enough to justify its study and estimation.

The *N* = 10,000 network estimated in Section 3.3 for demonstrating the effectiveness of EGP-based intervention is obtained from 100 SIR epidemics. In subtropical countries, there is typically one flu season each year. So if the estimation is based solely on flu, it would take 100 years to obtain the estimated network. In Singapore, because of the tropical climate and high population density, there is anecdotal evidence that epidemics of mild respiratory infections come around every one or two months. Assuming there are six epidemics a year, the estimation above will take a much shorter time of 17 years in this country. The main risk of using multiple infections to shorten the estimation time is the increased number of wrong estimates when two epidemics overlap. However, this is a problem that cell phone and GPS co-location can help to solve as well.

Finally, we highlight that the estimated network needs not to be very accurate to be *useful*. The results of Section 3.3 beautifully illustrate this, where targeted isolation based on an estimated network which is only 16.6% accurate is still very effective in mitigating epidemics (Figure 5a–b). This unexpected effectiveness of EGP applied on an inaccurately estimated network prompted us to better understand why this is happening. A deeper look at the preliminary study which we can visualize suggests that, while the predicted links are inaccurate, the nodes that emerged from the estimation are mostly hubs and are concentrated along the backbones of the infection network. Hence, removal of these nodes will effectively slow the advancing epidemic. Naturally, we expect even better results with a more accurate estimation.

### 4.2 Equal Graph Partitioning

Effective epidemic control measure is important for mankind as the problems associated with severe infectious disease can only increase in severity along with global population growth. To put this into the proper context, we highlight two most important features that characterize severe epidemics: (1) the large number of cases that emerges in relatively short time (surge in hospital load), and (2) the fast propagation of the disease as compared to treatment time and development of cure. We will discuss how the proposed mitigation method addresses these two problems.

For highly-infectious epidemics, the capacities of hospitals are stretched due to the large number of infected individuals appearing near the peak of the epidemic. Lack of medical attention and insufficient resources (medics, vaccines, and healthcare apparatus) might delay the recovery process and result to a higher number of casualties than need be. This is why it is important for an epidemic mitigation strategy to consider peak load reduction as a primary goal. Delaying the peak infection time, thus stretching the overall time scale of the epidemic, is also crucial. When dealing with a deadly epidemic caused by a new infection agent, chances are we would not have vaccine at hand, or even a vaccine development program. As development of vaccines and drugs for new diseases can only begin after cases have been confirmed and analyzed, the race against the epidemic to develop and administer such drugs would be inevitable. Delaying the progress of the epidemic will not only buy time for drug development, but will also give governments more time in general to implement other crisis handling measures.

Furthermore, any epidemic mitigation procedure can only be enforced after the epidemic has started and positive cases have been confirmed. Mitigation measures applied at the start of epidemic is ideal but may not be practical. Hence, good performance when applied late into the epidemic is critical for a mitigation method to be successful. Figure 5b hints at a the promising potential our proposed targeted strategy can offer in this regard, where significant reductions of the peak infection rate and the total number of infected people are observed even when applied late into the epidemic. However, the result also suggests that early implementation is critical in slowing down the epidemic.

Here let us also discuss the impact of the proposed strategy on privacy as a potential reason to object to its implementation. In a country like the US, this strategy is unlikely to be adopted no matter how well it is shown to work in simulation due to privacy concerns when we selectively isolate individuals according to EGP. However, the proposed strategy may find a more receptive audience in a country like Singapore, given that home quarantine orders have been issued by the Ministry of Health during the SARS and H1N1 outbreaks. Here we must stress that node removals as suggested by EGP need not be home quarantine or any other physical isolation practices. If early vaccine stocks are available but limited, the EGP nodes should be the ones to receive it, because of the role they play in disease transmission. We must nevertheless be wary that this procedure should not be used as a mean of discrimination.

Finally, we highlight a crucial difference of the proposed method to traditional strategies. Unlike conventional quarantine, this intervention involves isolating healthy individuals from the rest of the infection network. At first this might sounds absurd: “Why isolate healthy people and not the infected ones?” From network point of view, however, the reason is clear: by isolating these people we are intercepting the path of an ongoing epidemic. As comparison, it is a standard practice in controlling forest fire to cut or burn down trees ahead of advancing fire to contain it. For infectious diseases no one has imagined this intervention possible, but with an estimated infection network our proof-of-concept results are impressive. If all nodes on the entire estimated network are isolated, the results are even more impressive: for our *N* = 10,000 network with <*k*> = 10, we find propagating epidemics in only 60% of the simulations. In these propagating epidemics, the epidemic peak with intervention is less than 1/10 the epidemic peak without intervention, while the total number of infections with intervention is 1/3 that without intervention. Also, instead of peaking at *t* = 12 and ending at *t* = 20 without intervention, the epidemic peaks at *t* = 28 and ends at *t* = 60 with intervention. In terms of actual durations, removing all nodes on the estimated network stretches two-month epidemics to over half a year. However, about 1/4 of the population needs to be isolated, compared to only about 9% of the population for EGP intervention. A separate systematic study will be necessary to understand how effective and efficient this more aggressive intervention strategy can be.

In conclusions, we have presented in this work a proof-of-concept study of a novel epidemic mitigation method based on equal graph partitioning of the estimated infection network. We used computer simulations to study and show the effectiveness of the method. First, we followed the intuitive JGN method to generate artificial social network of various sizes but with roughly the same clustering characteristic to serve as the infection networks. We then simulated SIR epidemics on the artificial infection networks, recorded the incidences, and applied censorship with rates between 50% and 85% to mimic low reporting rates for mild infections. We then used the remaining incidence data to construct an estimate of the infection network using a modified JGN algorithm. We found that the estimation accuracy falls with increasing censor rate, average degree, and size of the network, but improves as more data from multiple epidemics are incorporated.

With the estimated infection networks at hand, we applied the Equal Graph Partitioning (EGP) algorithm to remove the smallest sets of nodes that will efficiently fragment the estimated networks. We then immunized the same set of nodes on the actual infection network before simulating subsequent epidemics. We compared the effectiveness of the targeted strategy to an untargeted method that randomly isolates the same number of nodes, and showed that the former outperforms the latter in decreasing and delaying the peak of the epidemics, as well as reducing the total number of infections. We also applied the proposed strategy at different times after the epidemic has started and showed that it is still effective in decreasing the peak infection rate and total number of cases even when applied late into the epidemic. In particular, we demonstrated that the EGP based strategy is surprisingly effective in mitigating epidemics even when the estimated network is not very accurate. Through visualizing small network, we find that in spite of the large number of wrongly estimated links, the nodes appearing in the estimated network are concentrated along the backbones of the actual network and thus play important roles in the disease transmission.

## Supporting Information

Video S1 **SIR epidemic on an artificial social network.** In this video, an SIR epidemic spreads through an artificial social network with *N* = 1,000 nodes, 1,584 links, average degree <*k*> = 3.168 and clustering coefficient *C* = 0.3928. The artificial social network is generated using the Jin-Girvan-Newman algorithm (Jin, Girvan, and Newman, 2001). The transmission probability used for this SIR epidemic is *p* = 0.80, and infected nodes recover after *t_R_* = 1 time step.
(WMV)

Video S2 **Another SIR epidemic on the artificial social network.** In this video, another SIR epidemic starts from a different seed on the artificial Jin-Girvan-Newman social network with *N* = 1,000 nodes, 1,584 links, average degree <*k*> = 3.168 and clustering coefficient *C* = 0.3928. The transmission probability for this SIR epidemic remains at *p* = 0.80, and infected nodes again recover after *t_R_* = 1 time step.
(WMV)

Video S3 **SIR epidemic on the EGP-immunized artificial social network. I.** In this video, an SIR epidemic spreads through the artificial Jin-Girvan-Newman social network after EGP immunization. The EGP immunization is based on the 475-node, 934-link network estimated over *S* = 100 SIR epidemics from the 1,000-node, 1,584-link network, using the algorithm described in the text, and a weight cutoff of *w_c_* = 7. Of the 934 estimated links, 738 are correct, giving an accuracy of 79.01%. The EGP procedure then identifies 67 separator nodes, and when these are removed, the immunized network has 1,359 links remaining.
(WMV)

Video S4 **SIR epidemic on the EGP-immunized artificial social network. II.** In this video, we start an SIR epidemic from a second seed on the EGP-immunized artificial Jin-Girvan-Newman social network. The original network consists of 1,000 nodes and 1,584 links, whereas the estimated network EGP is based on consists of 475 nodes and 934 links. 67 separator nodes identified by the EGP procedure are removed from the original network, giving an immunized network with 1,359 links.
(WMV)

Video S5 **SIR epidemic on the EGP-immunized artificial social network. III.** In this video, we start an SIR epidemic from a third seed on the EGP-immunized artificial Jin-Girvan-Newman social network. The original network consists of 1,000 nodes and 1,584 links, whereas the estimated network EGP is based on consists of 475 nodes and 934 links. 67 separator nodes identified by the EGP procedure are removed from the original network, giving an immunized network with 1,359 links.
(WMV)

Video S6 **SIR epidemic on the EGP-immunized artificial social network. IV.** In this video, we start an SIR epidemic from a fourth seed on the EGP-immunized artificial Jin-Girvan-Newman social network. The original network consists of 1,000 nodes and 1,584 links, whereas the estimated network EGP is based on consists of 475 nodes and 934 links. 67 separator nodes identified by the EGP procedure are removed from the original network, giving an immunized network with 1,359 links.
(WMV)
